# Crude extracts activity of three selected medicinal plants from the Venda region against some pathogenic organisms

**DOI:** 10.4314/ahs.v22i2.81

**Published:** 2022-06

**Authors:** Khumbelo Mabadahanye, Nolwazi L Bhembe, Ezekiel Green

**Affiliations:** Department of Biotechnology and Food Technology, University of Johannesburg, 55 Beit Street, Doornfontein Johannesburg 2028 South Africa; mabadahanyek@gmail.com, nbhembe@uj.ac.za, egreen@uj.ac.za

**Keywords:** Antimicrobial activity, crude extracts, medicinal plants, phytochemicals

## Abstract

**Background:**

The emerging of antimicrobial resistance has become a problem as it is threatening public health worldwide.

**Objectives:**

To extract crude extracts from three different medicinal plants, test activity against Mycobacterium smegmatis, Staphylococcus aureus and Escherichia coli and screen for phytochemicals of those that showed activity against the targeted bacteria.

**Methods:**

KirkiaacuminataOliv., Dichrostachyscinerea (L.) Wight &Arn. and MimusopszeyheriSond. plants were collected at Thengwe area, Mafukani village, Limpopo Province, South Africa. The plant materials collected were extracted using four solvents. Antimicrobial screening was accomplished using the agar well diffusion method and the crude extracts that showed activity against the targeted organisms were screened for phytochemicals using different tests.

**Results:**

With all solvents used for extraction, methanol had a greater yield of 14.1% from Dichrostachyscinerea crude extracts. Kirkiaacuminata and Dichrostachyscinerea were medicinal plants that inhibited Mycobacterium smegmatis and Staphylococcus aureus at the lowest concentration of 2.5 mg/ml and 1.25 mg/ml.

**Conclusions:**

The results from this study show that the selected medicinal plants are active against Mycobacterium smegmatis and Staphylococcus aureus and their pharmacological properties can be further analyzed for the development of new drugs.

## Introduction

The development of antimicrobial drug resistance (AMR) by microbial pathogens has become a serious problem as it threatens the public health globally 1. AMR defined as a complex process that normally involves the interaction between human, ecological and pathogen-related factors^1^. Antimicrobials have been used worldwide in animals and human medicine to improve the health and wellbeing 2. The use of antibiotics is growing worldwide in both industrial and developing countries where they end up being detected in surface waters, deposits and biota 3. The increase of AMR has brought to the 21^st^ century public health problems that is threatening the effectiveness of prevention and treatment of infections emerging caused by organisms (bacteria, parasites and viruses) that is no longer vulnerable to the medicines commonly used to treat those organisms 4. Rapid spread of resistant bacteria such as Staphylococcus aureus, Streptococcus pneumoniae, Neisseria gonorrhoeae, Mycobacterium tuberculosis, Klebsiellapneumoniae, Escherichia coli and Clostridium difficile to antibiotics has caused a serious problem worldwide due to high death rate 5,6,7. The resistance of Staphylococcus aureus to antibiotics was first detected in 1948, after the penicillin was introduced to treat deadly bacterial infections in 1943 7.

Mycobacterium tuberculosis have become the highest global burden due to the increasing reported cases of multidrug resistant tuberculosis (MDR-TB) making tuberculosis difficult to treat 7. TB is a topmost disease-causing death among persons living with human immunodeficiency virus (HIV) infection, which is about 40% of this population 8. The resistance of Mycobacterium tuberculosis to both isoniazid and rifampicin occurred between 3.6% and 18% of new and previously treated TB cases, distinct 8. The use of wrong treatment, comprising in prescription errors and low patient obedience or poor-quality drugs were associated with the development of resistant TB 4. In 2017, global incident cases of TB were estimated to be 10 million and an estimate of 1.3 million TB deaths occurred 8. The loss of lives due to AMR is estimated to be at about 50 000 lives in a year in the US and EU with 700 000 worldwide 9. The emergence of methicillin resistant Staphylococcus aureus (MRSA) infection has become problematic to public health sectors causing infections including meningitis, pneumonia, toxic shock syndrome, endocarditis, urinary tract, bone and joint infections 10. In Europe, more than 170 000 patients were affected with bloodstream MRSA infection with 5 400 deaths in 2007 10. The Centre for Disease Control and Prevention also reported that the bloodstream MRSA infections occurred in more than 80 000 patients with 11 285 deaths in the United States 10. It was also reported that the incidence of MRSA infection in most of African countries is increasing, though the rate is still 50% 10. Recent studies have identified that several genes are leading to the intrinsic resistance to a wide range of classes of antibiotics, including β-lactams, fluoroquinolones and aminoglycosides 11.

It was also reported that the resistant bacteria can be transferred to humans through food chain and wastewater from different operations such as hospital and pharmaceutical industries 12. Cox et al. 13, also reported that the misuse and mismanagement of antibiotics, low vaccination rates, poor sanitation, poor infection prevention and control practices are contributing to the increasing burden of drug-resistant infections in low- and middle-income countries (LMICs). According to Jindal et al. 12, World Health Organization (WHO) published The Global Strategy 2001 on how to fight against AMR, where different strategies were recommended such as educating patients, prescribers, dispensers from different levels and encouraging collaboration between industry, government bodies and academic for the development of new drugs and vaccines. It is recognized that teamwork is required across all resources settings worldwide to handle the issue of AMR 13.

The World Health Organization endorses the development of researches based on medicinal plants to identify new compounds that will help to treat diseases due to high resistance of microorganisms to antibiotics worldwide, especially in developing countries where Mycobacterium tuberculosis is prevalent 14. Medicinal plants have been the most important constituents of both traditional and conventional methods from centuries 15. According to recent reports, medicinal plants have a good potential to fight against diseases caused by microorganisms due to their secondary metabolites 15. Secondary metabolites are complex molecules that consist of many functional structures such as, flavonoids, polyphenols, coumarins and terpenoids^16^. The secondary metabolites of medicinal plants showed numerous pharmacological properties such as antioxidant, antibacterial, antiviral and antifungal^16^. This study aimed at extracting crude extracts from plant materials, to test for activity against the Mycobacterium smegmatis ATCC 607, *Staphylococcus aureus* ATCC 6571 and Escherichia coli ATCC 10536 strains and to screen for phytochemicals for those showing activity against the targeted organisms.

## Materials and Methods

### Study area, selection and collection of medicinal plants

**Figure 1:**
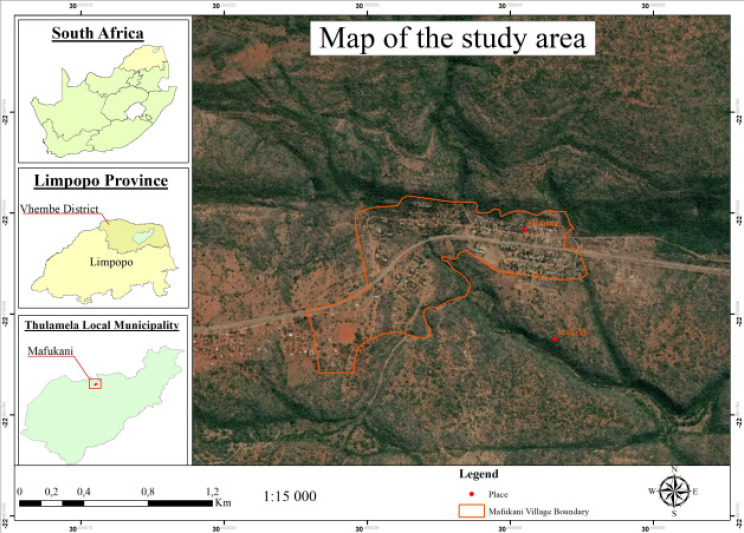
A map showing the study site where the plants were collected in Mafukani village, Limpopo Province of South Africa.

Three medicinal plants were collected at Mafukani village, Thulamela local municipality, Limpopo province, South Africa as shown in Figure 1. The geographical coordinates of the area are 22.6715°S, 30.5553°E. The plant samples were collected in their natural habitats in autumn (May 2018). Plant selection was done with the aid of a traditional herbalist, who has been treating TB infection with medicinal plants. The plants which were selected for this study were Kirkia acuminata Oliv., *Dichrostachys cinerea* (L.) Wight &Arn. and MimusopszeyheriSond. (Table 1). Part of the plant materials were sent to the University of Johannesburg herbarium for verification.

**Table 1:** Selected medicinal plants used in this study

Botanical Family	Species name	Voucher No	Vernacular name (V) = Tshivenda;	Life Form	Used plant parts	Aliment/s treated	References
Kirkiaceae	*Kirkia acuminata* Oliv.	KM0324	Mubvumela (V);	Tree	Barks Leaves	Cough, constipation, cholera, diarrhea, dysentery and wounds.	17,18,19
Fabaceae	*Dichrostachys cinerea* (L.) Wight & Arn.	KM0322	Muredzhe (V)	Tree	Seedpods	Tuberculosis, headaches, toothache, festering sores and wounds.	20,19,21
Sapotaceae	*Mimusops zeyheri* Sond.	KM0395	Mutaladzi (V);)	Tree	Leaves	Abdominal complaints. Diabetes mellitus, Candidiasis	22,23,19

### Plant material preparation

The different collected plant species parts were washed with distilled water to make sure that the dust and debris were removed and dried at room temperature for three weeks by spreading the parts of the plants on a flat surface. The samples were grounded into fine powder using an electric blender. All the samples were stored in zip lock bags in a dark place, until the extraction stage 24.

### Extraction of plant materials

The plant materials collected were extracted using four solvents (dichloromethane, ethyl acetate, acetone and methanol). Exhaustive extraction method was used to extract bark and ground leaves powder, with the increasing polarity from non-polar (dichloromethane) to polar (methanol). This was to guarantee that a wide range, polarity of compounds was extracted 25. The different plant samples were weighed, and each was extracted with a different volume of solvents depending on the amount of plant materials available for each plant species (Table 2). The ratio implemented was 1:10 26. The plant materials were soaked in the solvents, covered with constant shaking every 30 minutes for 6 hours and they were allowed to stand for 48 hours at room temperature as reported by Eze et al. 27. Mixtures were filtered using Whatman's No 1 filter papers and the solvents were removed under vacuum on a rotary evaporator at 40°C. The crude extracts were dried under the laminar floor for 3 to 6 days depending on the plant materials and weighed.

**Table 2:** The mass of dried plants weighed (g) and the solvents (ml) used for extraction

Plant species	Plant Part used	Powder weighed (g)	Solvents (ml)			
			DCM	ETAC	ACE	MET
*Kirkia acuminata*	Leaves	100	1000	1000	1000	1000
*Kirkia acuminata*	Barks	200	2000	2000	2000	2000
*Dichrostachys cinerea*	Seedpods	200	2000	2000	2000	2000
*Mimusops zeyheri*	Leaves	200	2000	2000	2000	2000

Solvents: Dichloromethane (DCM), ethyl acetate (ETAC), Acetone (ACE) and Methanol (MET)

Dried crude extracts were weighed in grams (g) and the total percentages extracted was calculated according to the method by Abarca-Vargas et al. 28 (Table 3).

**Table 3:** The table showing the average of solvents in grams

Solvents	Average (g)
Dichloromethane	2.92 ± 1.867
Ethyl acetate	0.89 ± 0.681
Acetone	7.57 ± 3.018
Methanol	19.12 ± 8.839

± Values of standard deviations

Equation (1):

%Yield = A/B x 100

Where A = the total weight of dried extracts obtained after drying and B = the total weight of ground plant material taken for each extraction process.

Microsoft Excel was used to calculate the average of four different solvents used and the standard deviations.

### Antimicrobial Screening

#### Preparation of microorganism

The microorganisms used were Mycobacterium smegmatis ATCC 607, Staphylococcus aureus ATCC 6571 and Escherichia coli ATCC 10536. The Middlebrook 7H9 broth was prepared supplemented with 0.2% glycerol and 10% oleic acid-albumin-dextrose-catalase (OADC) to culture Mycobacterium smegmatis strain, the bacteria was incubated for 24 hours at 370 in a shaker incubator^29^. Staphylococcus aureus ATCC 6571 and Escherichia coli ATCC 10536 were provided as cultures grown on blood agar plates. All these organisms were sub-cultured on Mueller Hinton agar and incubated for 24 hours at 35 ± 0.1°C in preparation for the agar well diffusion method^30^,^31^.

#### Preparation of plant extracts

Dried crude extracts were weighed, and each plant extract was dissolved in solvents (dichloromethane, ethyl acetate, acetone and methanol) used for extraction and 20% dimethyl sulfoxide as some of the plant extracts were not dissolving in 20% DMSO only. The stock solution of 100 mg/ml of plant extracts was obtained and the extracts were diluted into different concentrations ranging from 100 mg/ml to 0.3125 mg/ml 27,^32^.

#### Preparation of media for agar well diffusion method

A 200 ml of Mueller Hinton agar was prepared, autoclaved, poured in petri dishes and allowed to solidify at a room temperature. Wells of 5 mm diameter and 2.5 mm depth were bored in a solidified media using sterile cork borer 31.

#### Preparation of test microorganism

The sub-cultured microorganisms were adjusted for their turbidity using 0.5 McFarland standard (∼1.5 X 108 microorganisms/ml) 32.

Evaluation of antimicrobial activity of plant extracts

Test bacteria were inoculated on solidifying Mueller Hinton agar plates by streaking with a sterile cotton swab to obtain a confluent growth. A volume of 50 µl crude extracts (dichloromethane, ethyl acetate, acetone and methanol) were dispensed into the wells and pure dichloromethane, ethyl acetate, acetone methanol or 20% DMSO were also dispersed as negative controls. A volume of 50 µl of the 1.0 mg/ml rifampicin concentration equal to the volume of crude extracts was dispensed in the wells, which served as positive controls 31. Treated petri dishes were incubated for 24 hours at 37°. The zones of inhibition were measured using a ruler from the edge of the disks to the edge of the zone of inhibition 25.

### Phytochemical Screening

The crude extracts showing activity against M. smegmatis and *S. aureus* were selected and screened for phytochemical constituents using different screening methods (Phenols, Alkaloids Dragendorff's test, Flavonoids, Terpenoids Salkowski's test, Tannins Ferric Chloride test, Quinones, Saponins and Glycosides). Briefly, to test for Phenols a few drops of 3% ferric chloride solution were added into 1 ml of the plant extract. Formation of deep blue color was observed which confirms the presence of phenols 33.

For the Alkaloids Dragendorff's test, 0.5 ml of the plant extract, 2 ml of concentrated hydrochloric acid was added together with 1 ml of Dragendorff's reagent. Orange colored precipitate observed indicated the presence of alkaloids 34. To test for Flavonoids, 1 ml of the plant extract was added with 1 ml of sulfuric acid. An orange color was observed, indicating the presence of flavonoids 33.

The Terpenoids Salkowski's test was conducted as follows, 1 ml of the plant extract was treated with 2 ml of chloroform and 2 ml of concentrated sulfuric acid was added carefully along the sides of the test tube. A reddish brown colored layer was formed at an interface, indicating the presence of terpenoids 34.

The Tannins Ferric Chloride test was conducted in a test tube containing 5 ml of boiling distilled water, 0.1 ml of plant extract was added and boiled for an hour. After an hour, few drops of ferric chloride were added and allowed to stand for proper color development. A blue-black coloration observed, indicated the presence of tannins 35. To test for Quinones 1 ml of the plant extract was treated with 5 ml of hydrochloric acid and yellow color precipitate was observed, indicating the presence of known 33. The Saponins Foam test was conducted by mixing 0.5 mg of the plant extract with 2 ml of distilled water and shaking the test tube thereafter. A layer of foam formed and persisted for 10 minutes, indicated the presence of saponins 36. The Glycosides concentrated sulfuric acid (H2SO4) test was conducted with 5 ml of plant extract, 2 ml glacial acetic acid, one drop of 5% Iron III chloride (FeCl3) and sulfuric acid were added. A brown ring was observed,

## Results

### Plant Extraction

**Figure 2:**
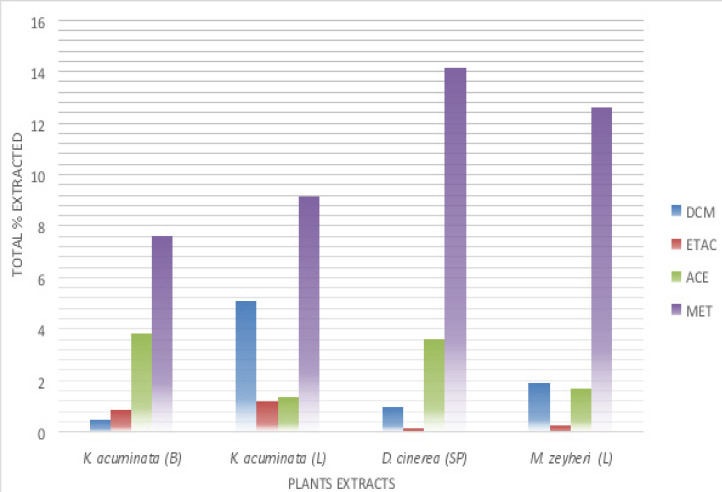
Total percentages of different plant species extracted with different solvents (DCM: Dichloromethane, ETAC: Ethyl Acetate, ACE: Acetone and MET: Methanol)

Plant materials were extracted using different solvents from non-polar to polar and different quantities of extracts were obtained (Figure 2).

Plant parts used: B: barks, L: leaf, SP: seedpods

The average of four different solvents used in the extraction were calculated as shown in Table 3.

### Antimicrobial Screening

The antimicrobial screening was carried out using the agar well diffusion method. Different diameters of the zones of inhibition were observed and measured in millimeters (Tables 4 and 5). The crude extracts were also screened against *E. coli* and no zones of inhibition were observed.

**Table 4:** Antimicrobial screening results of *Mycobacterium smegmatis*

Plant crude extracts	Zone of inhibition (in mm)								

	100 mg/ml	10 mg/ml	5 mg/ml	2.5 mg/ml	1.25 mg/ml	0.625 mg/ml	0.3125 mg/ml	- Control	Rifampicin
*K. acuminata* (B)									
DCM	7	9	7	-	-	-	-	-	30
ETAC	10	9	8	-	-	-	-	-	29
ACE	11	10	9	8	-	-	-	-	28
MET	7	8	7	-	-	-	-	-	30
*K. acuminata* (L)									
DCM	-	-	-	-	-	-	-	-	29
ETAC	-	-	-	-	-	-	-	-	30
ACE	10	9	-	-	-	-	-	-	27
MET	13	-	-	-	-	-	-	-	28
*D. cinerea* (SP)									
DCM	7	-	-	-	-	-	-	-	26
ETAC	7	7	-	-	-	-	-	-	29
ACE	16	10	8	-	-	-	-	-	27
MET	14	10	9	-	-	-	-	-	28
*M. zeyheri* (L)									
DCM	-	-	-	-	-	-	-	-	27
ETAC	8	7	-	-	-	-	-	-	30
ACE	12	11	10	-	-	-	-	-	28
MET	11	10	8	-	-	-	-	-	29

Extraction solvent: DCM: Dichloromethane, ETAC: Ethyl acetate, ACE: Acetone and MET: Methanol.

Plant parts used: B: barks, L: leaf, SP: seedpods

**Table 5:** Antimicrobial screening results of *Staphylococcus aureus*

Plant crude extracts	Zone of inhibition (in mm)							

	10 mg/ml	5 mg/ml	2.5 mg/ml	1.25 mg/ml	0.625 mg/ml	0.3125 mg/ml	- Control	Rifampicin
*K. acuminata* (B)								
DCM	-	-	-	-	-	-	-	40
ETAC	-	-	-	-	-	-	-	41
ACE	11	13	8	7	-	-	-	40
MET	12	10	9	-	-	-	-	40
*K. acuminata* (L)								
DCM	-	-	-	-	-	-	-	34
ETAC	-	-	-	-	-	-	-	41
ACE	16	10	7	-	-	-	-	40
MET	9	8	7	-	-	-	-	40
*D. cinerea* (SP)								
DCM	-	-	-	-	-	-	-	32
ETAC	-	-	-	-	-	-	-	44
ACE	17	12	9	7	-	-	-	37
MET	16	14	9	8	-	-	-	40
*M. zeyheri* (L)								
DCM	-	-	-	-	-	-	-	40
ETAC	-	-	-	-	-	-	-	37
ACE	11	9	-	-	-	-	-	36
MET	12	9	7	-	-	-	-	43

Extraction solvent: DCM: Dichloromethane, ETAC: Ethyl acetate, ACE: Acetone and MET: Methanol.

Plant parts used: B: barks, L: leaf, SP: seedpods

The phytochemical screening of selected active crude extracts was revealed in different chemical compounds shown in Table 6.

**Table 6:** Qualitative phytochemical analysis for ethyl acetate, acetone and methanol crude extracts from different medicinal plants

			Phytochemical constituents							
			
Plant species	Parts Used	Solvents	Phenols	Alkaloids	Flavonoids	Terpenoids	Tannins	Quinones	Saponins	Glycosides
*K. acuminata*	Barks	Ethyl acetate	+	+++	+++	+	-	+	+++	+
		Acetone	++	+	++	+++	+	++	-	+
*K. acuminata*	Leaves	Acetone	+++	+	++	+++	+++	+	+++	+
		Methanol	++	-	+	+++	+++	-	++	+
*D. cinerea*	Seedpods	Acetone	-	+	-	++	+++	+	+	+
*M. zeyheri*	Leaves	Methanol	-	+++	-	++	-	+++	+++	+
		Acetone	-	+	++	++	+++	+	+	+
		Methanol	++	+	+++	++	+++	+++	+	+

Key: - Absent, + Low concentration, ++ Moderate Concentration, +++ High Concentration.

## Discussion

Antibiotics have been used since the 20^th^ century to treat bacterial infections and some organisms have developed resistance to antibiotics 38. The microorganisms developed resistance through natural selection (Darwinian process) where microorganisms which are resistant to antimicrobial continue to flourish in its presence and the multi-drug resistance infections have emerged globally which is becoming a problem of public health 11,38. This necessitates urgent attention to screen for new anti-microbial compounds from medicinal plants that will be effective against the microorganisms causing infections. Medicinal plants have been used to cure different diseases from ages and can be used as a solution for this burden^39^. Extraction is the essential initial step in the analysis of medicinal plants, as it is important to extract ideal chemical compounds from the medicinal plant materials for further separation and characterization of compounds 40. In four solvents used in this study methanol was the best solvent to produce greater yields with 19.42 g, followed by acetone (7.57 g) and dichloromethane (2.92 g), while the low yield was obtained with ethl acetate (0.89 g) as shown in Table 3. The greatest yield of methanol was found in crude extracts of D. cinerea medicinal plant with 14.1% as shown in Figure 2. Masoko et al. 41 also found that methanol was the best extraction compared to other solvents. According to previous studies methanol is one of polar solvent that have been extensively used for extraction and was found to be more effective in extracting greater amounts of phenolic compounds from various plants 42. In another study, Boeing et al. 43, also found that methanol was the most effective solvent for recovery of antioxidant compounds; while acetone extracted lowest amount of antioxidant compounds because of the lower effectiveness of solvation and methanol molecules are a proton donor whereas acetone molecules are proton acceptors.

The results of the antimicrobial screening of 16 crude extracts tested against Mycobacterium smegmatis and *Staphylococcus aureus* appear promising while with *E. coli* there was no inhibition observed (which means the compounds were not effective against the organism). Acetone crude extracts of *K. acuminata*barks were able to inhibit M. smegmatis with a lowest concentration of 2.5 mg/ml and with 1.25 mg/ml in *S. aureus*. Kirkiaacuminata has been used as a source of traditional medicine in Central and Southern Africa to treat most common human diseases such as cough, cholera, abdominal pain, fever, diarrhea and toothache. It was reported that phytochemicals such as tannins, flavones, quercetin, gallic acid, phenols, acetic acid, propionic acid and isocoumarin were identified in *K. acuminata*and Kirkiawilmsii leaves, roots, seeds and twigs that can be responsible for a wide use of Kirkia species in traditional medicines 17,18. This also correlates with the results of phytochemical screening found in this study as they indicate that *K. acuminata*barks contain phenols, tannins and flavonoids that may be responsible for inhibiting both M. smegmatis and *S. aureus* in lower concentrations. It was reported that several medicinal plants which are rich in tannins have shown to contain antimicrobial activity against several microorganisms 44.

The acetone and methanol crude extracts of *D. cinerea* seedpods were also able to inhibit *S. aureus* in the lowest concentration of 1.25 mg/ml. The plant have been used across Africa and Asia to treat gonorrhea coughs, syphilis, headache, dysentery and sore eyes 45. Dichrostachyscinerea acetone extracts were found to have a higher concentration of tannins that can be responsible for activity against *S. aureus*. Based on this study results, it was observed that crude extracts of medicinal plants showed to be more effective against M. smegmatis and *S. aureus* with higher concentrations than in low concentrations, this is in concomitant with the work of Banso and Adeyemo^44^, who reported that the increase in antimicrobial effectiveness was observed with the increase in concentration of tannins isolated from *D. cinerea*.

In another study, Abreu et al. 46, reported on the resistance of *E. coli* to *D. cinerea*which was also observed in one of the medicinal plants selected in this study. According to Sheel et al. 36, plants contain different bioactive compounds that are found in the leaves, flowers, barks, seeds, fruits and roots that play an important role to cure different diseases. To the best of our knowledge, so far, no study has been done on *D. cinerea*seedpods to evaluate its antimicrobial properties as it ws first part of the plant to be used in the in vitro researches and has showed activity against bacteria.

## Conclusion

This present study shows the importance of medicinal plants and the effectiveness against some organisms, as it exhibited activity for both M. smegmatis and *S. aureus*. The K. acuminata barks and *D. cinerea*displayed good results in M. smegmatis and *S. aureus* and due to emergence of antibiotic resistance worldwide, these plants can be used for the development of new drugs. The plants are promising and can be used in future for therapeutic pur-poses.
